# Evaluating genomic tests from bench to bedside: a practical framework

**DOI:** 10.1186/1472-6947-12-117

**Published:** 2012-10-19

**Authors:** Jennifer S Lin, Matthew Thompson, Katrina AB Goddard, Margaret A Piper, Carl Heneghan, Evelyn P Whitlock

**Affiliations:** 1 Center for Health Research, Kaiser Permanente Northwest, 3800 N. Interstate Ave, Portland, 97227, OR, USA; 2 Department of Primary Care Health Sciences, University of Oxford, Oxford, OX1 2ET, UK; 3 Blue Cross and Blue Shield Association Technology Evaluation Center, Chicago, IL, 60601, USA

**Keywords:** Genetic/genomic, Test development, Diagnostic test, Prognostic test, Evaluation framework, Evidence-based decision making

## Abstract

The development of genomic tests is one of the most significant technological advances in medical testing in recent decades. As these tests become increasingly available, so does the need for a pragmatic framework to evaluate the evidence base and evidence gaps in order to facilitate informed decision-making. In this article we describe such a framework that can provide a common language and benchmarks for different stakeholders of genomic testing. Each stakeholder can use this framework to specify their respective thresholds for decision-making, depending on their perspective and particular needs. This framework is applicable across a broad range of test applications and can be helpful in the application and communication of a regulatory science for genomic testing. Our framework builds upon existing work and incorporates principles familiar to researchers involved in medical testing (both diagnostic and prognostic) generally, as well as those involved in genomic testing. This framework is organized around six phases in the development of genomic tests beginning with marker identification and ending with population impact, and highlights the important knowledge gaps that need to be filled in establishing the clinical relevance of a test. Our framework focuses on the clinical appropriateness of the four main dimensions of test research questions (population/setting, intervention/index test, comparators/reference test, and outcomes) rather than prescribing a hierarchy of study designs that should be used to address each phase.

## Introduction

The development of genetic and genomic tests is one of the most significant technological advances in medical testing in recent decades. A growing interest in “personalized” or “precision” medicine, commercial interests seeking return on investment, and limited regulatory oversight in many countries has resulted in increased availability of genomic tests. This environment has created a number of issues to determining the most appropriate point in time to adopt a new test in clinical practice which cannot be determined solely by availability, marketing, or regulatory approval.

Medical testing, in general, lags behind therapeutics in the understanding and application of rigorous evidence-based evaluation, and has particularly lacked a focus on patient outcomes. Because medical testing primarily provides information that may change patient management which indirectly affects patient outcomes, the direct impact on patient health can be difficult to assess. However, not only are diagnostic issues a greater source of medical errors and safety concerns than therapeutics, diagnostic testing also directs the majority of health care expenditure [1]. A focus on the net benefit of testing to patients (and a population of patients) is common to all stakeholders in medicine, and thus should be the unifying goal for any test evaluation framework.

Despite the importance of medical testing in patient management, the profit margins for the development of new tests are often low, compared to new pharmaceuticals, so there may be little incentive for diagnostic test developers to support clinical testing beyond that required for regulatory approval [2]. Generating and evaluating the evidence for genomic tests is further complicated by a set of terms and concepts unfamiliar to the majority of clinicians and their patients (Additional file 1). Genomic tests also have multiple applications in health care in addition to diagnosis, including screening, risk assessment, prognosis, and treatment selection (Table 1). Individual tests may have different roles within the same disease (e.g., Oncotype Dx [Genomic Health, Redwood City, CA] is used in both prognosis and treatment prediction of breast cancer), as well as across different diseases (e.g., KRAS testing is used to guide treatment decisions in both colorectal cancer and lung cancer).

**Table 1:** Multiple clinical roles of genetic tests in clinical practice

**Type**	**Purpose**	**Definition**	**Examples**
**Diagnostic**	Screening	Detection or exclusion of a characteristic or disease in asymptomatic persons	Fecal DNA to screen for colorectal cancer, SRY genotype to determine fetal sex in first-trimester
	Diagnosis	Rule in or rule out conditions in symptomatic persons	Lynch syndrome testing in patients with colorectal cancer, CFTR testing in patients with suspected cystic fibrosis, Factor V Leiden or prothrombin gene testing in patients with thromboembolic disease
**Prediction**	Risk assessment	Risk of future disease or morbidity from disease in people without the disease	Cardiogenomic profile in order to assess risk of future cardiovascular disease, BRCA testing in women at high risk for breast cancer
	Prognosis	Predicting outcomes in people with disease	Oncotype DX panel to assess prognosis in women with early stage breast cancer, BRCA testing in women with breast cancer
**Treatment**	Treatment selection or monitoring	Determine, predict, or monitor response and/or adverse effects of treatment	CYP2C19 gene to predict response to clopidigrel in patients with acute coronary syndrome or percutaneous coronary intervention (PCI)

There have been a number of models or frameworks designed to specifically structure the evaluation of genomic test development (Additional files 2 and 3) [3-16]. The majority of these frameworks build on the well-recognized ACCE (Analytic validity, Clinical validity, Clinical Utility, and Ethical, legal and social issues) framework [4-7,17,18], while others utilize the four or five phases of translational medicine (T1-T5) research [8-11]. The ACCE framework, as well as other models, are derived from a larger body of frameworks and criteria for the evaluation of medical tests in general (e.g. imaging, biomarkers) [18,19]. The majority of these frameworks are phased or tiered models that make a distinction between categories of evidence that address technical efficacy (analytic validity), diagnostic accuracy (clinical validity), and patient outcome efficacy (clinical utility) [19]. However, none of these diagnostic or prognostic models have been universally adopted by regulatory science agencies, health systems, or professional groups as the standard for the evaluating the evidence needed to inform decision-making around test regulatory approval, clinical use, reimbursement or guidelines implementation.

While these frameworks provide valuable insight into approaching the evaluation of genomic testing, each has important limitations. First, frameworks are too narrowly focused on a single testing role (e.g., screening), a particular clinical context (e.g., newborn screening), or one particular aspect or phase of evaluation (e.g., test validation). We believe that a single, inclusive framework would provide a common language across different stakeholders (e.g., clinicians, laboratorians, researchers, policy makers, test developers, and patients) despite their varying perspectives and needs. Second, frameworks such as the translational research models that specify phases of research from bench to bedside do not describe in sufficient detail the evidence needed within each phase of its development. From experience, we have found that general models lack specificity, are therefore difficult to operationalize and subsequently inconsistently applied. Third, some categories of frameworks are extremely comprehensive (involving detailed checklists or series of questions), and thus are better suited for identifying all possible research that might inform use of a genomic test without consideration of which research is necessary (and sufficient) before adoption in any specific clinical scenario. Together, these issues point to the critical need for a consistent and comprehensible framework for the efficient development and dissemination of genomic tests in order to facilitate informed decision-making.

Our aim is to describe a framework for assessing the evidence base for genomic tests (from discovery to clinical adoption) that builds upon existing published frameworks for evaluating genomic tests, without necessarily specifying any particular threshold of evidence for regulatory approval or clinical implementation. We anticipate this framework will be helpful in communicating where evidence for a particular diagnostic or prognostic test is missing, and inform the types of research needed before test implementation.

### Evaluation framework to define the evidence base for genomic tests

**Figure 1:**
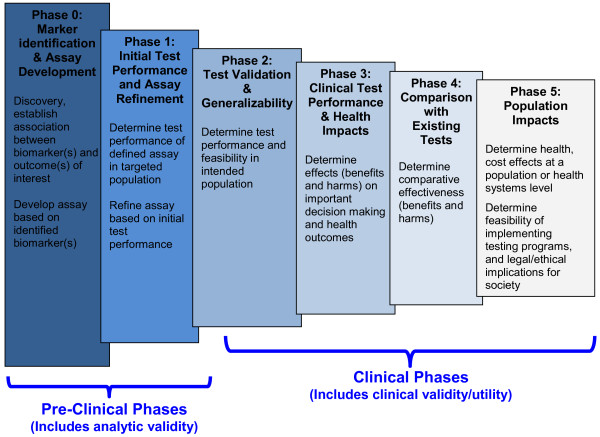
**Evaluation framework for genomic test development.** Tests should be evaluated within a given clinical context (i.e. specify disease or health condition, type of patient, proposed test role, desired outcomes, and current practice or clinical alternatives).

**Figure 2:**
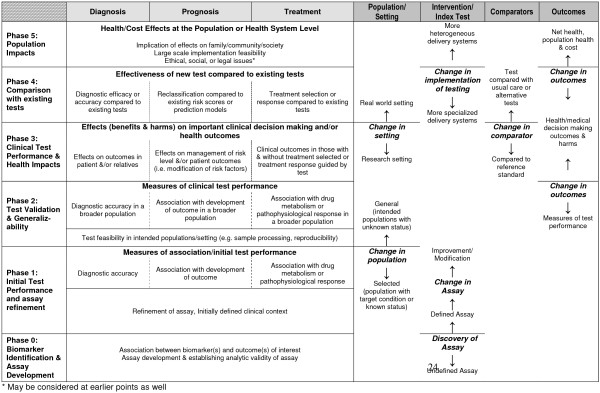
Framework for phased evaluation of new genetic tests in relation to proposed roles.

The proposed framework specifies six phases in the development of genomic tests and focuses on the important knowledge gaps that must be addressed before the tests can achieve clinical relevance (Figure 1 and Figure 2). Like some existing models, it parallels the four phases of drug development research [20], with the exception that it adds a Phase 0 for discovery and divides Phase 4 (post-marketing surveillance in drug development) into separate phases reflecting comparative effectiveness research and population impact. The framework incorporates the concepts and terminology used in previous frameworks distinguishing between technical efficacy (analytic validity), diagnostic accuracy (clinical validity), and patient outcome efficacy (clinical utility).

The phases of development are laid out from the "ground up" to illustrate the cumulative aspect of knowledge development that relies on solid foundations of pre-clinical evidence and proceeds through successively more applicable clinical studies (Figure 1). Although displayed linearly in this model, not all research in this arena proceeds in an orderly fashion. Test development research is highly iterative with possible modifications of the assay or test occurring at any phase of development. Intersecting with each phase of development (rows) are columns that specify the three main clinical roles of tests, namely ‘Diagnostic’ (including screening and diagnosis), ‘Predictive’ (including risk assessment and prognosis) and ‘Treatment’ (including selection and monitoring) (Figure 2). These intersections allow the user to visualize which aspects of development are shared across all test roles and which are unique to a particular role.

#### Phase 0: Biomarker identification and assay development

This phase encompasses the vast majority of genomic research, including agnostic genome-wide association studies (GWAS) and candidate gene approaches. An online catalog of GWAS published findings, for example, includes over 1000 publications and over 5000 disease-single nucleotide polymorphism (SNP) associations to date [21]. Maximum efficiency and success in assay development should begin with research targeting areas of clinical need, thereby ensuring that tests offer the greatest clinical value.

This phase also includes the early development of promising molecular biomarkers into genomic assays. There are multiple technical test aspects affecting assay development, including the selection of variant(s) to be evaluated, selection of a technology or platform for genotyping, and determination of sample and sample handling conditions. This phase should establish the initial analytic validity of the chosen assay that includes the basic technical performance of the assay (i.e., analytic accuracy, precision, and reproducibility). Ideally, this phase would result in a (well-defined) assay suitable for possible application in a specific disease or health condition that could be assessed in subsequent steps.

#### Phase 1: Initial test performance and assay refinement

A test’s initial clinical test performance is almost always examined in highly selected populations that are not representative of the intended population the test is to be used on. For example, tests may be conducted in persons with known disease compared to healthy controls, or in persons at very high risk for having the outcome(s) of interest (higher pre-test probability than the intended patients). For diagnostic tests, test performance is generally compared to a reference standard assay using measures such as sensitivity, specificity and predictive values. The approaches used to measure initial test performance, however, rely on the current state and accuracy of the science. For example, the diagnostic accuracy of a new genomic assay to measure cystic fibrosis transmembrane conductance regulator (CFTR) genotype would be compared with a functional assay, such as the sweat test, which is still the current standard for diagnosing cystic fibrosis [22]. The sweat test is not a perfect reference standard, but is better than mutation-based alternatives that lack demonstration of functional defects. However, lack of a true reference standard may result in misleading results if the newer test is actually superior [23]. For prognostic tests, initial assay performance is measured through strength of association between the assay result (e.g., presence of molecular variant[s]) and the development of the outcome (e.g., progression to a more advanced stage of disease, or development of disease complications). For tests that are used to select or monitor a treatment, measures of test performance may include association between the test result and an intermediate outcome, such as drug metabolism (e.g., serum level of endoxifen, the active form of tamoxifen after metabolism through CYP2D6), or pathophysiological response (e.g., tumor shrinkage, platelet aggregation). Using intermediate outcomes can also be misleading if these outcomes do not sufficiently discriminate between different treatment responses or are not robustly associated with true health effects in the patient [24]. If intermediate outcomes are used for efficiency at initial proof-of-concept phases, they should be sufficiently linked to patient outcomes through existing research.

Phase 1 also includes ongoing refinement of the assay’s technical performance. It is often necessary to refine the assay based on initial test performance by characterizing sample handling conditions or pre-analytic variables that result in superior performance of the test. For example, there may be changes in requirements for micro-dissection to isolate tumor tissue from contamination by normal tissue for a test of somatic mutations, or changes in the buffer conditions to stabilize a sample during transfer to a reference laboratory. This iterative process of initial clinical validation and assay refinement can sometimes lead to significant changes in the assay. As such, it is important for test development research to be explicit about the similarities and differences in iterative “versions” of assays. Fecal Deoxyribonucleic acid (DNA) testing, for example, has undergone multiple iterations during its development as a potential screening tool for colorectal cancer. As a result, the currently available assay compared to previously test versions (different biomarkers and different technologies) [25], which has resulted in discrepancies amongst different clinical recommendations [26,27].

#### Phase 2: Test validation and generalizability

Although, studies in this phase often use the same outcome measures as studies in Phase 1 (i.e., measures of diagnostic accuracy), they establish the performance of the test in populations in which the test is intended to be used. The differences in study design and population between Phase 1 and Phase 2 often lead to differences in test performance [28]. In the development of a genomic assay to screen for colon cancer, for example, the initial test performance in Phase 1 would establish the assay's ability to differentiate known cases from controls. In Phase 2 however, the performance of the assay is evaluated in a sample of all patients eligible for colorectal cancer screening, which allows determining the assay's performance in the intended population and its feasibility under less idealized conditions. Phase 1 research (initial test validation) showed that early versions of fecal DNA testing had very high sensitivity (around 90%) but Phase 2 research conducted in a screening population showed much lower sensitivities (around 25 to 50%), and also revealed problems with test implementation in a clinical setting. These Phase 1-Phase 2 discrepancies led to substantial alterations in subsequent versions of the fecal DNA assays [29].

#### Phase 3: Clinical test performance and health impacts

While previous phases focus solely on the diagnostic or prognostic ability of these tests, research in Phase 3 addresses the clinical impact and net benefit (i.e., tradeoff between benefits and harms) to patients from using the test compared to not using the test. Outcomes for phase 3 can include the impact of testing on patient management and treatment decisions (e.g., increased cancer surveillance, monitoring for progression or recurrence, or choice in treatment regimen) and subsequent patient health outcomes (e.g., morbidity, mortality, adverse effects and quality of life). Established diagnostic accuracy or risk prediction (clinical validity) does not necessarily translate into improved health outcomes (clinical utility). This lack of effect on health outcomes may occur because a test may provide information which does not (or cannot) lead to changes in treatment or management options. Even if management options exist, they may not be effective in improving health outcomes. Alternatively a genomic test may identify a genotype with incomplete or variable penetrance, or the harms (or other tradeoffs) of testing outweigh or mitigate the clinical benefits.

One well known example of genomic testing with established clinical utility is BRCA1/2 testing (e.g., BRACAnalysis, Myriad Genetics Inc., Salt Lake City, UT). There has been significant research in evaluating patient outcomes from bilateral prophylactic mastectomy to prevent breast cancer in carriers of BRCA 1/2 mutations, demonstrating improved survival and quality of life [30]. In contrast, genomics tests for Factor V Leiden (FVL) or prothrombin mutations in patients with idiopathic venous thromboembolism (VTE) (or in their family members) displays good clinical validity, but lacks evidence of clinical utility. Although an increased risk for VTE recurrence (in patients) or VTE occurrence (in family members) is well-established, knowledge of mutation status is unlikely to significantly benefit patients or their family members [31]. Thus, this phase’s key goal is to demonstrate that the test result triggers a clinical action that leads to improved health outcomes.

#### Phase 4: Comparison with existing tests

Research in this phase aims to answer the question of whether the test result provides added clinical value above and beyond other clinical information already available, or compared to existing tests. Whilst genomic tests can be novel tests for conditions for which no tests currently exist, it is always important to consider the existing standard of care: whether the new test performs better than any existing tests, or whether the new test has equal diagnostic/prognostic ability, but is less invasive or less expensive. For example, clinicians need to know whether an assay for cytochrome P450 (CYP) 2C19 variants is superior to existing platelet functioning testing in predicting response to clopidogrel in persons with acute coronary syndrome (or undergoing percutaneous coronary intervention). This issue of “comparative effectiveness” is paramount for genomic tests designed for risk prediction or prognosis. Ten new biomarkers have recently been identified that are strongly associated with breast cancer risk in older women at the genome-wide significance level [32]. However, the performance of a risk prediction model using these biomarkers, as measured by the area under the curve (AUC), is only slightly better than risk models that use four traditional non-genetic risk factors. Thus, the new biomarkers do not provide sufficient additional information beyond traditional risk factors to warrant adoption, despite a highly statistically significantly association. Genetic profiles for diabetes [33] and cardiovascular disease [34] have similarly demonstrated a failure to provide a significant improvement over models using only traditional risk factors and do not offer other advantages in terms of cost, accessibility, or acceptability.

#### Phase 5: Population impacts

The final phase of this framework evaluates the new test’s population impacts, including its implications for the family, community, or society as a whole (including ethical and/or legal issues). For example, an accurate method has been developed to determine fetal gender by detection of the sex-determining region Y (SRY) genotypes in maternal blood during the first trimester, which should only be present if the fetus is male [35]. Even if this test has moved far along the phases of development, for many societies the use of this test for non-medical purposes would have major ethical and moral issues. The cost of new tests also has major implications for multiple stakeholders, so this phase might include health economic evaluations. For example, while it is theoretically possible to screen all newly diagnosed colorectal cancer patients for mutations in Lynch Syndrome genes, the cost of germline testing would far outweigh the costs saved from identifying individuals with mutations and implementing programs for early detection or prevention of cancer [36-38]. Instead, other approaches to identify high-risk individuals for testing have been recommended based on the clinical characteristics of the patient or the results of preliminary laboratory screening tests [27,39,40].

### Strengths of the proposed framework

This framework provides a common language and benchmarks that can be used by diverse stakeholders. This is important because while test developers may have expertise in particular genetic tests, these tests are likely to be evaluated increasingly by those with less-specialized knowledge as a test advances through phases of development. As such, we have incorporated general principles familiar to those doing research in medical testing generally (diagnostic and prognostic) as well as those doing research in genomic testing (e.g., ACCE and laboratory-based validation principles). We have also tried to use shared terminology and concepts that correlate with both medical testing and pharmaceutical development.

This framework does not prescribe a hierarchy of study designs that should be used to answer the questions in each phase because the risk of bias within any given study (considering both design and implementation) is more important than the type of study per se. Multiple study designs may be possible within each of our proposed phases. Instead, our framework focuses on the transitions in the four main dimensions of test research questions (population/setting, intervention/index test, comparators/reference test, outcomes) as the main organizing principle to categorize the hierarchy of evidence for test development/performance across the “phases” of research (Figure 2). Phases 0 and 1 focus on the establishing the index test (I). The pivotal transition from Phase 1 to Phase 2, involves evaluating the clinical performance of the assay in populations (P) for whom it is designed. Phase 3 represents a further substantial change in outcomes (O) of interest from the clinical accuracy of testing (Phases 1 & 2) to the net impact of the test on patient health outcomes (clinical utility). In Phase 4 the pivotal transition is the comparison (C) to existing tests or clinical data used to inform clinical decision making about what test works better (or has less harm), or the added value of a test to managing patient care. Phase 5 represents another pivotal change in outcomes (O), with a shift in focus to population level outcomes, often including cost, and societal level implications of testing beyond the individual patient.

Finally, our proposed framework incorporates different clinical roles of genetic tests across the multiple phases of evaluation. As such, it avoids the “silo” approach of having multiple different frameworks for each different role of tests and highlights shared research aims across different test roles. For example, demonstrating an association between genetic variant and clinical outcome of interest is common to all potential future uses of genetic tests in Phase 0. In contrast, in Phase 1 the key outcomes for a genetic test depend on its intended use as a screening/diagnostic test versus prognostic or pharmacogenomic test. In Phase 3, studies of different clinical applications may differ in both study population and outcomes. For example, a study of BRCA1/2 testing for risk assessment (prediction) in asymptomatic women might evaluate how well the test classifies a woman’s risk of developing breast cancer over a specific time period, which could modify screening recommendations or prophylactic management strategies. A study of the same test’s use in determining appropriate treatment options in women who already have breast cancer, on the other hand, would focus on the test's ability to distinguish between those who did and didn’t respond to specific treatments [41]. Highlighting these shared and separate research aims may allow test developers and researchers to focus on potential clinical uses of new genetic tests.

### Implications of proposed framework to relevant stakeholders

We have synthesized across many existing models[4-7,17-19] to describe a unifying framework that allows stakeholders to comprehend the state of the evidence (and evidence gaps) for any given genomic test. This is especially important for genomic tests because their development and dissemination is both rapid and iterative, rather than an orderly one-way path from discovery to adoption in clinical practice. While a phased evaluation scheme allows for efficient, ethical research planning, in reality, it is not a rigid progression in medical testing or drug development. An orderly progression may characterize the early pre-clinical stages (Phases 0 and 1 in our framework), but it is less common beyond these stages [19]. While it may not be necessary (or realistic) for test developers to conduct research that moves through the phases one level at a time, this framework allows decision makers to understand the existing evidence in the context of the entire evidence landscape hierarchy.

As genomic testing is increasingly disseminated into clinical practice, clinicians and their patients will encounter these tests on a regular basis. As a result, we anticipate the framework will be useful in communicating to clinicians and their patients, what evidence is available for a genomic tests and/or what evidence would be needed before considering using it for routine clinical use. Through using the framework it should be quite apparent that, for example, the latest media report of a new gene-disease association is only at the start of the evidence-gathering (Phase 0), and thus deserves little of the precious clinical time available for determining current patient care options.

Our framework also allows different stakeholders to specify different thresholds for decision-making, depending on their perspective and particular needs (e.g., for exploration, further development or discontinuation, regulation, clinical uptake, insurance coverage, dissemination, practice guideline development, or marketing). Regardless of where different stakeholder may choose to set their threshold, the framework usefully organizes evidence into two main stages: pre-clinical (Phases 0 and 1) where a test may be in various phases of development but not ready for clinical adoption and clinical phases (Phases 2 through 5), whereby increasing certainty of clinical impact is investigated. It is important to articulate these initial pre-clinical Phases 0 and 1 in a genomic testing framework, because often discovery research (e.g., agnostic GWAS) or early/initial validity testing (e.g., case control studies in patient with known disease and healthy controls) pushes the early adoption of genomic testing into clinical practice. Although Phase 2 research may establish clinical validity in the relevant patient populations it does not translate directly into improved patient outcomes and therefore may be an adequate threshold for some stakeholders (e.g., test regulators), but not others (e.g., payers, health care delivery systems).

Medical laboratory testing regulation is much more limited than, for example, the regulation of new drugs. In both the United States (US) and Europe, regulation is based heavily on technical performance and demonstration of some clinical relevance, but not on patient outcomes [42-44]. In the US, the vast majority of genomic tests in the US are laboratory-developed tests (LDT), not actively regulated by the U.S. Food and Drug Administration (FDA), but instead regulated by the Centers for Medicare and Medicaid Services for the overall quality of laboratory testing under the Clinical Laboratory Improvement Amendments (CLIA), which addresses implementation of testing largely after the clinical adoption [45]. The Secretary's Advisory Committee on Genetic Testing (SACGT) has recommended that the FDA be responsible for the review, approval, and labeling of all new genetic tests that have moved beyond the basic research phase (i.e., Phase 0), focusing on the evaluation of analytical validity and clinical validity (i.e., Phases 1 and 2) as well as on claims made by the test developer about its clinical utility (i.e., Phases 3 through 5) [46].

Our proposed framework could help unify perspectives and shared understanding in the same way that the four phases of drug development allow a clearly understood benchmarking process for approval and usage of new pharmaceuticals. If this framework were incorporated into the FDA's developing “evidence-based regulatory science” approach, regulatory agencies could be explicit on what an appropriate level of evidence (taking into consideration different populations, comparator tests, and outcomes) might be for a given assay and what constitutes a similar enough (vs. new/different) assay. Other stakeholders (e.g., payers or health systems) could similarly reflect upon and be explicit about their threshold of evidence before uptake, as different bodies will inevitably have different perspectives.

Our suggested framework is intended to facilitate ongoing discussion and developmental activities among manufacturers, test researchers, systematic reviewers, regulators, and other policy-makers, as well as to facilitate understanding among clinicians and patients. We believe our framework is broad enough to be applied to a wide variety of genomic testing, however, we realize that the complexities and rapid advances within genomics (next generation sequencing) and related fields (proteomics, metabolomics) will necessitate re-evaluating and tailoring our framework over time. If our effort reflects a useful synthesis of existing frameworking efforts to date, it may allow more consistency in definitions for terms and concepts going forward and provide a platform for future collaborative efforts.

### Conclusions and future directions

The field of genomics is one of the potentially most important developments in health care in the 21^st^ century, holding out the promise of revolutionizing medicine towards a more personalized approach. However, we believe that in order to achieve their potential, and to avoid inappropriate use, genomic tests need to be much more broadly understood, and dialogue between different types of users needs to be facilitated. Our proposed framework improves upon the efforts of others, and offers unique features, including specifying in more detail the clinical questions and changes in research focus that accompany the clinical development of a genomic test. If this framework is applied generally, it will help users understand the state of the science for a given genomic application and to articulate their own clinical thresholds for evidence that may be required for a test to be adopted. This, in turn, could reduce confusion, minimize the possibility for inappropriate use, and enhance innovation.

## Abbreviations

ACCE: Analytic validity, Clinical validity, Clinical utility, Ethical/legal/social issues; AUC: Area under the curve; CFTR: Cystic fibrosis transmembrane conductance regulator; CLIA: Clinical Laboratory Improvement Amendments; CYP: Cytochrome P450; DNA: Deoxyribonucleic acid; FDA: U.S. Food and Drug Administration; FVL: Factor V leiden; GWAS: Genome-wide association study; KRAS: Kirsten rat sarcoma oncogene; LDT: Laboratory-developed tests; SACGT: Secretary's Advisory Committee on Genetic Testing; SNP: Single nucleotide polymorphism; SRY: Sex-determining region Y; US: United States; VTE: Venous thromboembolism.

## Competing interests

The authors declare that they have no competing interests.

## Authors’ contributions

JSL, Designed and developed the framework with examples and drafted the article, and worked on final revisions; MT, designed and developed the framework with examples and drafted the article, and worked on final revisions; KAB, developed the framework with examples, revised the article for intellectual content; MAP, revised the article for intellectual content; CH, revised the article for intellectual content; EPW, Developed the initial idea for the framework, developed the framework with examples, revised the article for intellectual content. All authors read and approved the final manuscript.

## Pre-publication history

The pre-publication history for this paper can be accessed here:

http://www.biomedcentral.com/1472-6947/12/117/prepub

## Supplementary Material

Additional file 1 **PDF; Glossary of terms.** Key terms used are defined. Click here for file

Additional file 2 **Search strategy and article flow.** Describes the search method we used to conduct our abbreviated systematic review. The number of abstracts and articles reviewed throughout the process are also indicated. Click here for file

Additional file 3 **Existing frameworks used to evaluate the evidence base for genomic testing.** The main frameworks used in the field of genomics are identified and described in detail. Click here for file
