# Supplementary material for: Evaluating genomic tests from bench to bedside: a practical framework
Source: BMC Med Inform Decis Mak. 2012 Oct 19;12:117. doi: 10.1186/1472-6947-12-117 (PMC3538070; doi:10.1186/1472-6947-12-117)
Supplement: Additional file 1 — PDF; Glossary of terms. Key terms used are defined. [file 1472-6947-12-117-S1.pdf]

Additional file 1: Glossary of terms

Term	Definition
Analytic validity	An assay's ability to accurately and reliably measure the genotype (or analyte) of interest.
Assay vs. Test	An assay is an analysis conducted to verify the presence (and amount) of a substance.<br>A genetic test is a laboratory assay that is used to identify a particular genotype or set of genotypes, within a specific context (i.e. for a particular purpose, in a particular population, for a particular condition).
Biomarker or Genetic marker	An identifiable sequence of DNA with enough variation between individuals that its inheritance and co-inheritance with alleles of a given gene can be traced. An allele is one of two or more versions of a gene. A biomarker can also refer to the biochemical material, RNA or protein, resulting from the expression of a gene.
Clinical validity vs. utility	Clinical validity is a test's ability to accurately and reliably predict the clinically defined disorder or phenotype of interest. Clinical validity can be expressed by measure of associations (between genotype and outcome of interest) or test performance characteristics, such as sensitivity, specificity, positive and negative predictive value [4].<br>Clinical utility is the evidence of improved measureable clinical outcomes and the test's usefulness and value it adds to patient management decision-making, compared with current management without genetic testing.
Genetic vs. Genomic	Genetics is the branch of science concerned with the means and consequences of transmission and biological inheritance, often focused on rare, mendelian single gene diseases.<br>Genomics concerns the genome, which is all DNA within an organism or cell, including both chromosomes within the nucleus and DNA in the mitochondria; the field tends to focus on profiles of lots of genes.
Genome wide association study (GWAS)	A method of searching the genome for single nucleotide polymorphisms that occur more frequently in people with a particular condition than in people without it.
Germline vs. somatic mutation	A germline mutation is present in germ cells (eggs or sperm) and can be passed to subsequent generations.<br>Somatic mutations are alterations in DNA that occur after conception, and can occur in any cell of the body except the germ cells.
Mutation vs. Variant	A mutation is any alteration in a gene from its natural state; may be disease causing or benign and can be rare or new (de novo).<br>A variant is an alteration in a gene that has established itself in a population and can evolve over time.
Single nucleotide polymorphism (SNP)	DNA sequence variation in which a single nucleotide in the genome differs between individuals in a species.
