# Supplementary material for: Evaluating genomic tests from bench to bedside: a practical framework
Source: BMC Med Inform Decis Mak. 2012 Oct 19;12:117. doi: 10.1186/1472-6947-12-117 (PMC3538070; doi:10.1186/1472-6947-12-117)

Additional file 2: Search strategy & article flow

Search Strategy

We searched MEDLINE for the following words: ((genetic* or genomic*) adj2 test*) OR ((genetic* or genomic*) adj2 screening) AND/OR (hierarchical adj (model* or approach* or evaluation*)) AND/OR (phrased adj (approach* or evaluation*)) AND/OR (phased adj (approach* or evaluation*)) AND/OR framework* (325 hits, February 2011). We then manually checked the reference lists of identified papers for other related publications. After reviewing the 325 abstracts from our search and five articles from outside sources, we identified 31 full text articles of interest to review. After independent dual review of full text articles, we included (and abstracted) 15 articles.

Article Flow

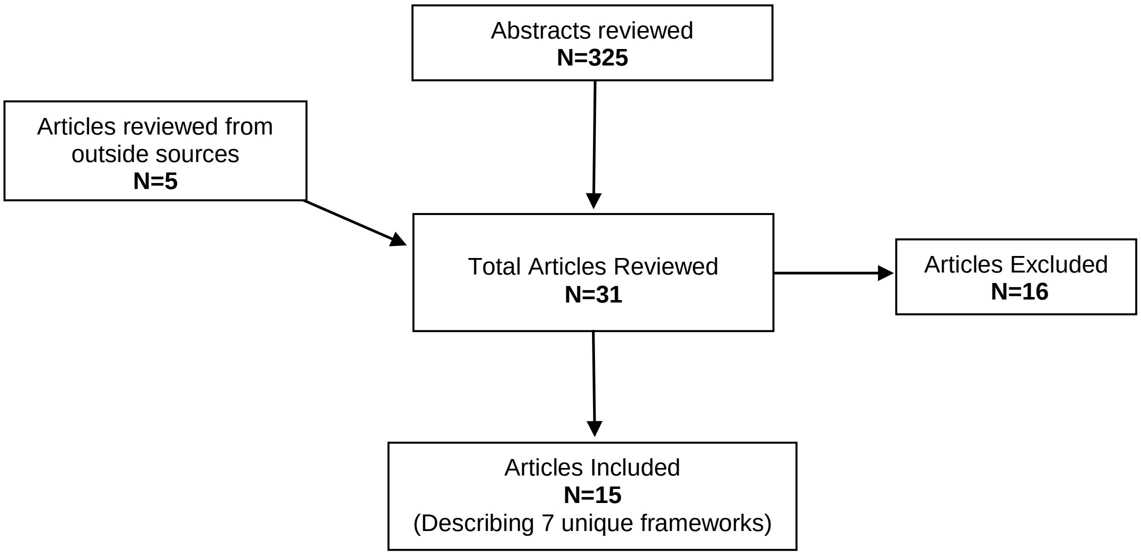

Supplement: Additional file 2 — Search strategy and article flow. Describes the search method we used to conduct our abbreviated systematic review. The number of abstracts and articles reviewed throughout the process are also indicated. [file 1472-6947-12-117-S2.pdf]
