# Supplementary material for: Evaluating genomic tests from bench to bedside: a practical framework
Source: BMC Med Inform Decis Mak. 2012 Oct 19;12:117. doi: 10.1186/1472-6947-12-117 (PMC3538070; doi:10.1186/1472-6947-12-117)
Supplement: Additional file 3 — Existing frameworks used to evaluate the evidence base for genomic testing. The main frameworks used in the field of genomics are identified and described in detail. [file 1472-6947-12-117-S3.pdf]

Additional file 3: Existing frameworks to evaluate evidence base for genomic testing

Author, year Institution	Framework	Aim	Primary audience	Description of framework or added value to original framework
Haddow and Palomaki, 2003<br><br>CDC	ACCE	Develop an assessment process for genetic testing	Systematic reviewers, researchers and policy-makers	Original ACCE model. Considers 4 assessment components (analytic validity, clinical validity, clinical utility, and ethical, legal, and social issues), each with a standardized set of questions.
Burke 2007<br>Sanderson 2005<br><br>PHG Foundation		Incorporate health quality measures in the evaluation of genetic tests & associated services	Researchers	Distinguishes between two components within the concept of clinical validity.<br>Expands the components of clinical utility using established measures of health care quality.<br>Proposes new preliminary domain to describe the context in which test will occur.
Teutsch 2009<br><br>EGAPP working group		Outline hierarchy of data sources and study designs to assist in quality rating and assessing evidence	Systematic reviewers and policy-makers	Presents specific methodological guidance on hierarchies of evidence.
Goodman 2009<br><br>CMTP		Improve the quality of evidence supporting the use of genetic tests by providing useful standards	Test developers, researchers, and policy-makers	Presents specific methodological guidance on types of study design.<br>Introduces new concept of “added clinical value”.<br>Introduces the need to describe the pre-analytic factors.
Mattocks 2010<br><br>EuroGentest Validation Group		Develop scheme for validation & verification of molecular genetic tests for diagnostic use	Clinical and laboratory researchers (scientists)	Focuses on analytic and clinical test validation.<br>Presents specific methodological guidance on what measures to use (and when), and how to report them.
Hornberger 2012<br><br>Cedar Associates LLC		To develop a set of criteria to evaluate gene-based laboratory developed tests used for clinical decision making	Test developers, researchers, investors, regulators, and policy-makers	LTD-SynFRAME builds on ACCE by specifying criteria within clinical validity (on study design, population, clinical meaningfulness, and statistical significance) and adding a category of economic validity (evaluating the economic implications of the test).
Khoury 2007<br>Agurs-Collins 2008	Stages of translational research (T1-T4)	Create framework for translational research for moving promising genomic applications into practice for public health benefit	Researchers, policy-makers, funding agencies, industry, community groups	Attempts to integrate and apply existing frameworks to genetic testing (i.e., NIH translation roadmap (T1-T4), ACCE framework, and phases of drug development).
Rosenkotter 2010		Create framework to evaluate new genomic technologies using traditional public health assessment instruments	Researchers and policy-makers	Attempts to integrate and apply existing public health assessment tools (i.e., Health Technology Assessment, Health Needs Assessment, Health Impact Assessment) to genetic testing and the phases of translational research (T1-T4).

Additional file 3: Existing frameworks to evaluate evidence base for genomic testing

Author, year Institution	Framework	Aim	Primary audience	Description of framework or added value to original framework
Khoury 2010	Stages of translational research (T1-T4), cont.	Describe contributions of epidemiology to translational research	Researchers and policy-makers	Adds T0 phase “scientific discovery”; description of application of epidemiologic methods to each phase of translational research.<br>Highlights role of knowledge synthesis at each stage. Emphasis on non-linear nature of test development research.
Andermann 2010		To make the multiple factors influencing the policy-making process more explicit & transparent	Policy-makers, public health experts, clinicians, basic scientists, & patient group representatives	3 parts: 1) genetic screening programs as having three integrated levels (laboratory testing, clinical services, and program management); 2) policy-making process, 3) description of broad & complex arena in which genetic screening policy-making occurs. Acknowledges cyclical process. Encompasses broad perspective of multiple stakeholders.
Arar 2011<br><br>GAPPNet	Genome-based Knowledge Management in Cycles Model (G-KNOMIC)	Propose framework for knowledge management that considers knowledge synthesis, evaluation, dissemination, and utilization as inter-related and essential elements to enhance the translation of genomic applications	Stakeholders across the public health sector, including researchers, practitioners, policy-makers, and educators	4 step cycle: 1) knowledge synthesis: aggregate existing research and evidence using explicit and reproducible methods to identify, appraise, and synthesize relevant studies, 2) knowledge evaluation: seeks to understand and measure accuracy, reliability, validity and utility of genomic-based services, 3) knowledge implementation: carrying out plans for providing genomic-based services, and 4) utilization: uptake and adoption of new genomic-based services by consumers and providers. Acknowledges cyclical process. Encompasses broad perspective of multiple stakeholders.
Botkin 2011		Develop process for evaluating proposed newborn screening programs in order to improve the evidence base and research structure from which programs are recommended	Researchers and policy-makers	4 stages of research: 1) projects that seek to determine whether early detection & intervention can improve clinical outcomes, 2) whether population-based screening results in net benefits to affected children compared to alternative methods of detection, 3) relative costs of a population-wide screening program, 4) evaluate established programs on an ongoing basis.

Additional file 3: Existing frameworks to evaluate evidence base for genomic testing

Author, year Institution	Framework	Aim	Primary audience	Description of framework or added value to original framework
Rousseau 2010<br><br>International Federation of Clinical Chemistry and Laboratory Medicine	Genetic testing evidence tracking tool (GETT)	Develop tool for systematic collection, presentation and summary of available peer-reviewed data and evidence related to genetic tests destined for clinical use	clinicians and policy-makers	72 specific items grouped in 10 themes: 1) overview of the disease (epidemiology and genetics); 2) diagnostic tools, 3) quality improvement programs; 4) clinical utility, 5) screening or diagnostic strategies; 6) impacts on health care system, 7) psychological and social aspects of the analysis, 8) ethical and legal aspects of the analysis; 9) synthesis; 10) research priorities. Provides a template and format for the presentation of evidence. Provides more explicit description of the reference standard and alternatives. Explicitly addresses impacts on health care systems (e.g., cost effectiveness and accessibility). Addresses availability of quality improvement or proficiency testing programs. Addresses availability and accessibility of professional services, healthcare, and follow-up, expertise and training.
Sun 2011<br><br>ECRI	Analytic Frameworks	To clarify a comprehensive framework or set of frameworks for evaluating genetic tests	systematic reviewers, policy-makers	Analytic frameworks modified from EGAPP frameworks, separate framework for each testing scenario (diagnosis in symptomatic patients, disease screening in asymptomatic patients, prognosis assessment, treatment monitoring, pharmacogenetics, risk/susceptibility assessment, germline-mutation related testing). Visual depiction of "chain of evidence" and relationship between population, test under consideration, subsequent interventions, and outcomes (intermediate outcomes, patient health outcomes, and harms). Useful for operationalizing evidence reviews.
